# Retracted: Physical Exercise and Patients with Chronic Renal Failure: A Meta-Analysis

**DOI:** 10.1155/2024/9862479

**Published:** 2024-02-21

**Authors:** BioMed Research International

*BioMed Research International* has retracted the article titled “Physical Exercise and Patients with Chronic Renal Failure: A Meta-Analysis” [1], due to concerns regarding the validity of the data. Following the publication of a Letter to the Editor [2], investigations were conducted into the relevance of studies included in Table 2. The authors responded to provide a revised table, however, this was deemed unsatisfactory by the Editorial Board and the article is therefore being retracted due to concerns with the validity of the data and conclusions.

The authors did not respond to our correspondence regarding these concerns.
